# Evaluation of whole‐body MR to CT deformable image registration

**DOI:** 10.1120/jacmp.v14i4.4163

**Published:** 2013-07-08

**Authors:** A. Akbarzadeh, D. Gutierrez, A. Baskin, M.R. Ay, A. Ahmadian, N. Riahi Alam, KO Lövblad, H. Zaidi

**Affiliations:** ^1^ Department of Medical Physics and Biomedical Engineering Tehran University of Medical Sciences Tehran Iran; ^2^ Medical Imaging Systems Group Research Center for Molecular and Cellular Imaging, Tehran University of Medical Sciences Tehran Iran; ^3^ Division of Nuclear Medicine and Molecular Imaging Geneva University Hospital Geneva Switzerland; ^4^ Research Center for Nuclear Medicine Tehran University of Medical Sciences Tehran Iran; ^5^ Division of Neuroradiology Geneva University Hospital Geneva Switzerland; ^6^ Geneva Neuroscience Center Geneva University Geneva Switzerland; ^7^ Department of Nuclear Medicine and Molecular Imaging University of Groningen, University Medical Center Groningen Groningen The Netherlands

**Keywords:** image registration, deformable model, PET/MRI, PET/CT, attenuation correction

## Abstract

Multimodality image registration plays a crucial role in various clinical and research applications. The aim of this study is to present an optimized MR to CT whole‐body deformable image registration algorithm and its validation using clinical studies. A 3D intermodality registration technique based on B‐spline transformation was performed using optimized parameters of the elastix package based on the Insight Toolkit (ITK) framework. Twenty‐eight (17 male and 11 female) clinical studies were used in this work. The registration was evaluated using anatomical landmarks and segmented organs. In addition to 16 anatomical landmarks, three key organs (brain, lungs, and kidneys) and the entire body volume were segmented for evaluation. Several parameters — such as the Euclidean distance between anatomical landmarks, target overlap, Dice and Jaccard coefficients, false positives and false negatives, volume similarity, distance error, and Hausdorff distance — were calculated to quantify the quality of the registration algorithm. Dice coefficients for the majority of patients (>75%) were in the 0.8–1 range for the whole body, brain, and lungs, which satisfies the criteria to achieve excellent alignment. On the other hand, for kidneys, Dice coefficients for volumes of 25% of the patients meet excellent volume agreement requirement, while the majority of patients satisfy good agreement criteria (>0.6). For all patients, the distance error was in 0–10 mm range for all segmented organs. In summary, we optimized and evaluated the accuracy of an MR to CT deformable registration algorithm. The registered images constitute a useful 3D whole‐body MR‐CT atlas suitable for the development and evaluation of novel MR‐guided attenuation correction procedures on hybrid PET‐MR systems.

PACS number: 07.05.Pj

## I. INTRODUCTION

Since the infancy of digital image processing techniques, image registration has widely been used in medical applications. The quest for automatic or even semiautomatic alignment of various medical images containing identical or different information spurred the development of robust multimodality image registration algorithms. The manifold applications of image registration is beyond the scope of present study; however, pointing to a number of noticeable applications in medicine is helpful. Image registration plays an undeniable role in image comparison procedures such as treatment verification carried out by comparison of pre‐ and postintervention images,^1^, ^2^ subtraction of ictal and inter‐ictal SPECT images,^3^, ^4^ bone growth monitoring on X‐ray or ultrasound time series,^(5)^ tumor growth monitoring on series of MR scans,^(6)^ monitoring response to treatment in PET,^(7)^ and temporal differencing for motion detection.^(8)^ Other wide scopes for image registration are statistical atlas generation^(9)^ and atlas‐based segmentation,^(10)^ and attenuation correction on hybrid PET/MR systems.^(11)^ This later reference reports on an MR‐guided attenuation correction method relying on an atlas consisting of registered MR and CT images of several subjects.

In clinical oncology, the need for integration of anatomical and functional information for diagnosis, staging, and therapy planning has pushed medical imaging technology into the area of multimodality and multiparametric imaging.^12^, ^13^, ^14^ Multimodality imaging systems combine the advantages of both anatomical imaging modalities (e.g., CT, MRI, ultrasound, portal imaging) and functional imaging modalities (e.g., SPECT, PET, fMRI, MRS). With the advent of multimodality imaging, image registration became an essential component of both clinical and research applications and is increasingly attracting the interest of the medical imaging community.^(15)^

Recently developed hybrid PET/MRI systems provide the combination of anatomical–functional and even functional–functional information.^13^, ^16^, ^17^, ^18^ Although hybrid PET/MRI provides highly accurate hardware based coregistered images, the lack of suitable and efficient motion correction techniques and the need for MR‐guided attenuation correction make software‐based image registration an essential component of this technology.

The procedure used for atlas generation consists of two major parts: image registration and conversion. A large number of image registration algorithms were proposed varying in dimensionality, nature of transform, modalities involved, and optimization procedure. Owing to the various features associated with the procedure, different classifications were presented.^(19)^ Regardless of the type of registration, the evaluation and validation of image registration algorithms play a pivotal role in the whole process. Image registration depends on the geometry of both the target image (the fixed image on which the second image is registered) and the source image (the moving image that is registered on the target image). Therefore, introducing a thorough concept that guarantees the accuracy of the registration process in whole‐body imaging proved to be a difficult task. However, some general guidelines were proposed for validation of image registration, particularly for the thoracic imaging.^20^, ^21^ Validation of image registration algorithms usually follows a sequence of measurements using computer‐generated anatomical models,^(22)^ physical phantoms^23^, ^24^ or images of patients or volunteers.^24^, ^25^ Although validation based on phantom studies holds a high precision owing to the accurately known transformation parameters of warped phantoms as well as the inverse transform which is used as ground truth for evaluation, it is not inclusive since realistic patient geometry is hardly considered. The issue becomes more complex in the case of intermodality registration. On the other hand, validation based on clinical images is prohibitive in terms of time and availability of expert evaluators. In other words, either for localization of anatomic landmarks or segmentation of organs, expert knowledge (as reference) is necessary, which deters investigators from following this approach.

In this study, we describe a deformable intermodality (MR to CT) image registration approach with the aim to exploit it for atlas generation in the context of MR‐guided attenuation correction. The atlas is made up of aligned whole‐body CT and MR images of 28 patients. The performance of the 3D intermodality registration technique is evaluated using clinical images through manually defined anatomical landmarks and segmented organs.

## II. MATERIALS AND METHODS

### A. Image registration

Image registration is the process of finding a spatial transformation from one image or domain to another according to a given set of constraints. This constraint could be a special kind of similarity measure. Consequently, the task is to optimize the transformation parameters based on the candidate similarity measure. Our registration process includes two main steps. The first step allows the achievement of preliminary alignment through a rigid transform to roughly align the target and source images. The rigid transform with six parameters consists of a sequence of translation and rotation transforms. The second step consists of a nonrigid registration procedure by means of the B‐spline deformable transform. This registration strategy is based on methods described by Rueckert et al.^(26)^ The B‐spline transformation is a free‐form deformation described by a cubic B‐spline function defined on a uniformly spaced control point grid (independent of the image data) covering the image being registered. B‐spline registration requires initial control points which were selected uniformly to produce a 3D grid. During the registration process, transform parameters are calculated for this grid; then the whole 3D image is transformed in each loop. As such, the registration process is fully automated and was carried out without user intervention for all studies.

The registration of whole body images was carried out using elastix,^(27)^ a command line driven program based on the Insight Toolkit (ITK) registration framework (open source; National Library of Medicine).^(28)^ The registration parameters were selected based on our own assessment of the various possible options and recommendations provided by the developers of elastix based on their experience and feedback from a large community of users. We used parameters corresponding to lung registration study, since the lungs were the most problematic regions.^(29)^ The first tests demonstrated that this is a good candidate for our task, but some changes were needed to achieve optimal performance following in‐depth evaluation of the impact of the various parameters involved. The three most important steps of the registration procedure:

1. The rigid registration was performed using a multiresolution registration approach at five different levels, from a level 16 times lower in resolution to full original resolution.
2. The elastic registration was carried out using three resolutions, from eight times lower to twice the original resolution.
3. The total number of iterations for each of the two registrations was about 8000.

We set higher pyramid levels for rigid registration since large and rough transform steps are required. Besides, when we increase pyramid levels and/or iterations in the B‐spline registration, the resulting image tends to deviate from the target owing to the fact that higher pyramid levels and/or number of iterations lead to overdeformation of the resulting image. The normalized mutual information (NMI) metric was considered as similarity measure, but other measures available in elastix could have been used. However, better performance was reported when using the normalized correlation coefficient (NCC) for intramodality registration and the NMI for intermodality registration metrics. In agreement with observations made by other users, a slightly better performance was achieved using the NMI for intermodality registration because this metric is able to manage contrast scale differences introduced by different modalities. We therefore used NMI metric with 32 bins, 4096 samples for elastic, and 5000 samples for rigid registration.

A steepest descent optimizer was chosen to optimize the similarity measure. This optimizer uses the gradient vector to move through the cost function until an optimum is found. To determine the best step size and learning rate which is not trapped inside local minima, the whole process was repeated several times. For each resolution, a specific step size was assigned to the optimizer. For the gradient descent, the step is given by:

(1) $$Step=\frac{a}{{\left(A+k+1\right)}^{a}}$$

where *k* is the iteration number. The parameters a,A,k, and α are summarized in Table 1. This formula was used by Klein et al.^(30)^ and was judged to be appropriate for our registration procedure. For obvious reasons the step size is large in the beginning and gradually becomes smaller at higher iterations. The image transformation consists of two steps: (i) transformation in physical space, and (ii) resampling (from physical space to voxel space). The B‐spline transform is equivalent to the generation of deformation fields where a deformation vector is assigned to every point in physical space. The deformation vectors are computed using 3rd order B‐spline interpolation from the deformation values of points located on a uniform grid. The uniform grid spacing was eight voxels which accounts for around 30,000 control points. It has been shown that a smaller number of randomly selected control points speeds up the process without affecting negatively the optimization process.^(30)^ Therefore, 5,000 points were randomly selected in each iteration. Conversely, the resampling step requires interpolation to calculate the intensity values of the transformed image. In order to speed up the resampling process, for each iteration, a 1st order B‐spline method was used to interpolate the moving image, whereas a 3rd order B‐spline method was used for the final iteration.

The registration process was fully automatic, taking less than 30 min on Intel Core 2 Quad processors (Intel Corporation, Santa Clara, CA). Once the process is finished, the software writes an ASCII file containing the optimized parameters for each transformation, namely six parameters for the rigid registration and tens of thousands (variable depending on the dataset) for the elastic registration. This ASCII file is used by other utilities (Transformix) to deform the source image to produce the final registered image.

**Table 1:** Summary of the parameters required to determine the step size for the optimizer

*Type*		*Rigid*					*Elastic*				
Resolution (in number of pixels)		16	8	4	2	1	8	4	4	2	2
No of iterations		4096	2048	1024	512	256	4096	2048	1024	512	256
Gradient descent parameters	*a*			4000			2000	7000	20000	20000	30000
	*A*			50					50		
	α			0.6					0.602		

### B. data acquisition

Whole body X‐ray CT and MR images were acquired for 28 (17 male and 11 female) patients. The patients were randomly selected from the clinical database (age between 18 and 81 years). The weight and height ranges differ between male (height: 164 to 179 cm; weight: 59–95 kg) and female (height: 148 to 168 cm; weight: 38–88 kg) patients. It should be emphasized that in most of the cases, patients’ disease state did not affect the registration process, except a few cases which were excluded when the presence of large metallic implants deteriorated the overall image quality.

CT images were acquired for attenuation correction of PET/CT studies on the Biograph HireZ scanner (Siemens Healthcare, Erlangen, Germany). CT scanning was performed using 120 kVp tube voltage and CARE Dose (Siemens) tube current modulation which sets the tube current depending on body thickness. Each image volume contains between 300 and 400 transaxial slices with a resolution of 512×512pixels. The voxel dimensions are $1.367\times 1.367\times 2.5\;{\text{mm}}^{3}$. Three‐dimensional MR image volumes were acquired using the Achieva 3T X‐series (Philips Healthcare, Andover, MA) MRI subsystem of the hybrid PET/MRI system installed at Geneva University Hospital.^(31)^ MRI image volumes contain approximately 180–200 axial slices (320×320pixels). The voxel dimensions are $1.875\times 1.875\times 6\;{\text{mm}}^{3}$. A 3D T1‐weighted spin echo pulse MR sequence was acquired with 200 frequency encoding steps, as well as phase encoding steps at 4.7 ms intervals (TR: 4.07 ms; TE: 2.3 ms; flip angle: 10°). The time interval between MR and CT data acquisition varied between approximately from 30 to 90 minutes.

Higher resolution with finer sampling would have been desirable for optimal image registration but, since this study was conducted retrospectively, we had only access to low resolution whole‐body MR images (with rough voxel resolution to increase SNR) used for attenuation correction and anatomical localization. Smaller voxel sizes will provide noisier images of even poorer quality, which might worsen the registration process.

### C. Evaluation

The most relevant issue in correlative imaging is the ability to locate the same anatomical features in coregistered images. In other words, a perfect registration algorithm completely aligns all corresponding points and objects of the source and target images (in our case organs or landmarks of human anatomy). Hence, the validation step requires accurate segmentation of specific organs, as well as localization of anatomic landmarks. One of the prevalent reference or ground truth for segmentation of organs and localization of landmarks is expert knowledge. Despite its inherent limitations and reproducibility, expert knowledge is the still the most widely used reference for evaluation of image registration techniques. Consequently, we can measure three different kinds of metrics for registration evaluation: point, surface, and volume. For each patient, 16 anatomical landmark spatial coordinates were defined on both CT and MR images by a radiologist (Table 2).^32^, ^33^ The Euclidean distance between these points in the two datasets was calculated as the registration error. In order to evaluate volume and surface discrepancies, the whole‐body, along with three key organs (brain, lungs, and kidneys) was segmented. The segmentation algorithm consists of a combination of manual and active contour models^(34)^ (snakes) using an interactive program which facilitates the task of manual segmentation and initial contour definition. The active contour model is used to accelerate the segmentation process but is prone to leak in some regions, wherein manual intervention is required. In addition, kidneys were not easily distinguished on the low‐resolution MRI and in some cases on CT where manual segmentation was carried out. However in three cases, the kidneys were left out, since they were not distinguishable on MRI, even by experienced radiologists.

**Table 2:** Statistical characteristics of registration errors (in mm) corresponding to predefined anatomical landmarks for grouped analysis of 28 clinical studies presented in Fig. 2. Δ is calculated by a statistical test on groups which shows confidence interval for 95% degree of significance. Its range is shown by notches in the plots

	*Minimum*	*1st Quartile*	Median±Δ	*3rd Quartile*	*Maximum*
Apex of right lung	0.33	3.87	7.74±2.63	12.58	20.51
Apex of left lung	0.95	5.29	7.46±1.75	11.07	17.61
Liver dome	0.00	8.14	14.01±4.32	22.45	28.92
Anterior right liver border	0.84	9.14	14.61±3.29	20.04	32.40
Anterior left liver border	3.78	7.31	11.52±3.20	17.91	33.32
Lateral liver border	2.39	8.26	11.98±3.03	18.29	25.97
Posterior liver border	0.00	5.32	9.51±2.58	13.85	18.78
Inferior liver border	0.00	5.29	7.61±2.37	13.15	22.07
Right adrenal	0.00	5.46	8.43±3.68	17.63	33.30
Celiac trunk	0.00	4.88	6.32±3.77	17.37	30.47
Upper tip of right kidney	0.00	4.85	6.87±1.77	10.70	18.73
Upper tip of left kidney	2.28	4.71	8.19±1.78	10.59	17.91
Lower tip of right kidney	0.74	3.89	7.24±2.17	11.09	18.76
Lower tip of left kidney	0.00	5.56	10.21±3.43	16.89	27.11
T10 vertebra	0.00	3.54	9.25±3.66	15.64	24.49
L5 vertebra	4.55	8.48	13.01±3.48	19.99	34.11

Various strategies were proposed to measure the discrepancy between volumes and surfaces of corresponding segmented volumes of source and target images. These strategies can be classified into three different categories as discussed by Klein et al.:^(25)^ volume overlap, volume overlay errors, and surface distance measures.

#### C.1 Volume overlap

These are the most often used methods to evaluate image registration techniques. Basically these methods are based on measurements of union, intersection, and subtraction of segmented volumes. They roughly quantify the fraction of source (S) and target (T) volume labels that agree or disagree. A good description of these methods, including multiple labels, fractional labels, and weighted labels, is given in Crum et al.^(35)^ The more accurate the registration, the closer these quantities approach the unity. For this reason, these methods belong to the same group.

Target overlap can be calculated over a set of r regions (regions that were labeled through segmentation):

(2) $$TO=\frac{S_{r}\cap T_{r}}{T_{r}}$$

Mean overlap (Dice coefficient) is a figure of merit that quantifies the intersection between source and target labels divided by the mean volume of the two regions:

(3) $$DC=2\frac{S_{r}\cap T_{r}}{\left|S_{r}\right|+\left|T_{r}\right|}$$

The Dice coefficient^(36)^ is a special case of Kappa statistical coefficient.^(37)^ A possible interpretation of the results of this quantification is given below:^(38)^

- Poor agreement→less than0.2
- Fair agreement→0.2to0.4
- Good agreement→0.6to0.8
- Excellent agreement→0.8to1.0

Union overlap (Jaccard coefficient ‐ Tannimoto coefficient) is a figure of merit that quantifies the intersection between source and target labels divided by the union of both regions:

(4) $$JC=\frac{S_{r}\cap T_{r}}{S_{r}\cup T_{r}}$$

The Jaccard coefficient^(39)^ (also known as Tannimoto coefficient^(40)^) is treated separately from the Dice coefficient; however, one should note that both are related by the following formula:

(5) $$DC=\frac{2.JC}{\left(1+JC\right)}$$

#### C.2 Volume overlay error

Following the previously described volume overlay methods that quantify the agreement between source and target regions, we can also compute the disagreement by making the hypothesis that the target volume is the truth we must achieve and the source volume is a tentative to achieve this truth. The false‐negative (FN) value can then be defined as the proportion of target volume that is incorrectly not covered by the source volume:

(6) $$FN=\frac{\left|T_{r}\backslash S_{r}\right|}{T_{r}}$$

Inversely, the false‐positive (FP) error can be defined as the proportion of source volume incorrectly not covering the target volume:

(7) $$FP=\frac{\left|S_{r}\backslash T_{r}\right|}{S_{r}}$$

Volume similarity is a figure of merit that measures the similarity between target and source volumes regardless of the fact that the volumes are superimposed. Therefore, it is not technically a quantification of registration accuracy. However, it provides information about which registration method gives a closest source volume to the target volume.

(8) $$VS=2\frac{\left|S_{r}\right|-\left|T_{r}\right|}{\left|S_{r}\right|+\left|T_{r}\right|}$$

#### C.3 Surface distance measures

The above‐mentioned figures of merit do not explicitly assess boundary discrepancies between source and target regions. The distance error (DE) is a well‐defined quantity to measure the boundary mismatch between the source and the target.^(25)^ This distance is equal to the minimum distance from each boundary point of the source region ($S_{\text{Bp}}$) to the entire set of points of the target region ($T_{B}$), averaged across the N boundary points:

(9) $$DE=\frac{1}{N}\sum_{p=1}^{N}\text{min}dist(S_{Bp},T_{B})$$

The most frequently used discrepancy measure is the Hausdorff distance (HD), which measures the maximum distance one would need to move the boundaries of the source region to completely cover the target region.^(35)^ Formally, ${\text{HD}}_{S\to T}$ from the source region (S) to the target region (T) is a maximum function defined as:

(10) $$HD_{S\to T}=\underset{s\in S}{\max}\{\underset{t\in T}{\min}\{d(s,t)\}\}$$

#### C.4 Implementation

A set of classes were written in C++ object‐oriented language to implement the above‐described image registration validation metrics, taking advantage of ITK classes for image I/O operations and well‐defined image data structure. Figures of merit for volume discrepancy measurement were calculated by counting the voxels of union, intersection, and subtraction of each region. The calculation of surface distance required a faster algorithm owing to the high complexity of finding the minimum Euclidian distance of adjacent voxels for all points on the surface. As a result, the distance map for each region was generated and used to find the surface discrepancies.

## III. RESULTS

**Figure 1:**
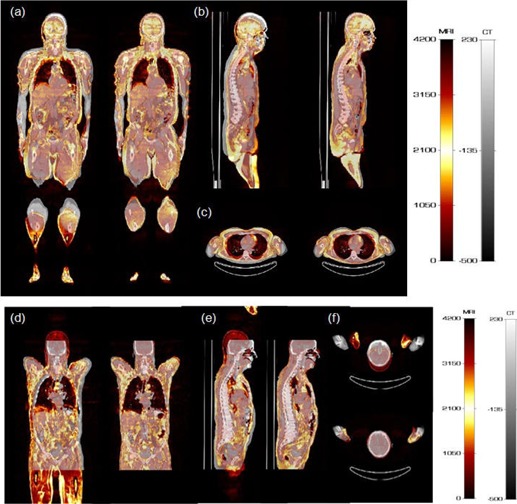
Representative clinical studies of two patients showing 3D fused MRI and CT images before and after image registration: (a) and (d) coronal, (b) and (e) sagittal, and (c) and (f) transaxial views. For each image pair, left and right panels represent the same image before and after registration, respectively.

**Figure 2:**
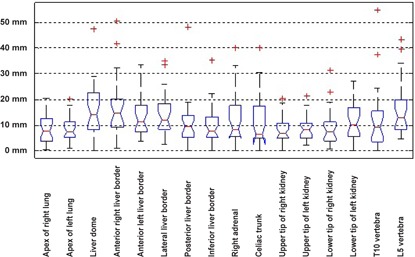
Box and whisker plot of registration error for manual evaluation performed by an experienced physician using different anatomic landmarks.

Figure 1 shows two representative clinical studies before and after registration. The misalignment between MRI and CT images at the level of the arms and head is commonplace in whole body imaging. It is visually obvious that the images were matched satisfactorily after registration. The results of evaluations for the considered patient population in terms of point‐to‐point evaluation of the deformable registration procedure are shown in Fig. 2 using box and whisker plot. The box shows the median (red horizontal line) and the lower (Q1) and upper (Q3) quartiles (defined as the 25th and 75th percentiles). The red plus sign in the plots indicates the outliers. Outliers in our plot include dubious results which are beyond 1.5 times the interquartile range

(Q3–Q1). A possible origin of dubious results is the failure of the image registration process in cases of poor MR image quality so that the organs are not distinguishable enough. In these cases, the cost function fails to correctly guide the optimizer. The whiskers show the maximum and minimum of population after elimination of outliers. The box and whisker plots not only indicate the quantitative characteristics of the samples under study, but also hold useful features for comparison of different groups of samples. The notches in each box depict the confidence interval for the median at 95% of confidence degree. Two analogous medians with confidence intervals that overlap are not significantly different. It can be seen that for the majority of data points (above 75%), the error is under 20 mm. The detailed statistical analysis results are reported in Table 3.

**Figure 3:**
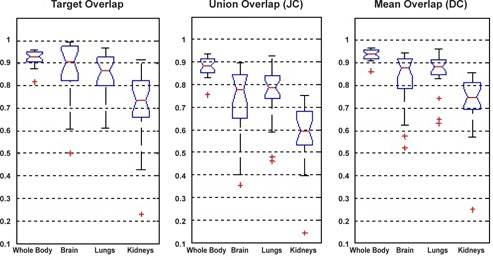
Box and whisker plot for registration evaluation using volume overlap metric.

**Figure 4:**
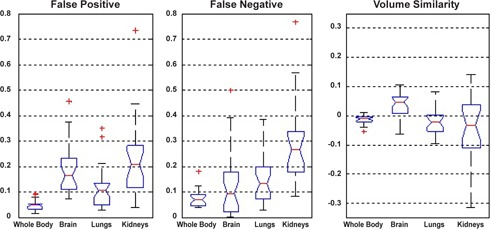
Box and whisker plots for volume overlay errors: (a) false positive, (b) false negative, and (c) volume similarity.

Figure 3 shows box and whisker plot for volume overlap of various organs. As mentioned earlier, the comparison was performed for the whole body and for three specific organs (brain, lungs, and kidneys). The plot indicates some outliers considered as dubious results. The false positive, false negative, and volume similarity plots for the 28 patients show an excellent alignment (error under 0.1) for whole body registration (Fig. 4). False negative is the fraction of the target image volume that is not intersected by the source image, which is under 0.35 for the majority of patients. On the other hand, the false positive is a fraction of source image that is not intersected by target image. The majority of patients have false‐positive value under 0.3 for all evaluated organs.

**Figure 5:**
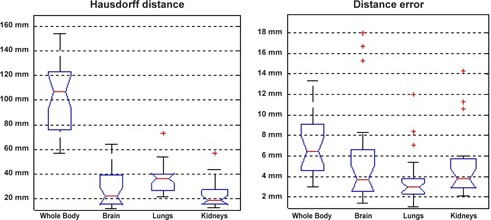
Box and whisker plots for the surface distance measure for the whole body, brain, lungs, and kidneys: Hausdorff distance (a) and distance error (b).

**Figure 6:**
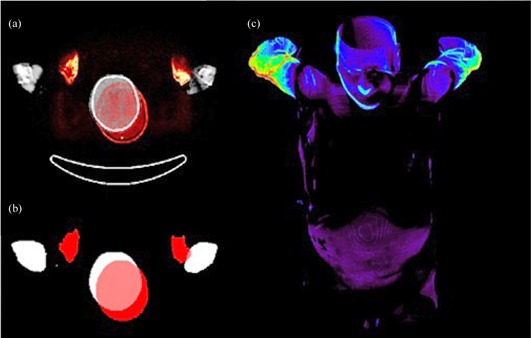
The misalignment of the arms and head is demonstrated on the overlaid CT and MRI images (a). The same images are shown (b) after segmentation and labeling (MR, red; CT, white). 3D view of distance map for whole body for the worst case study (c).

**Figure 7:**
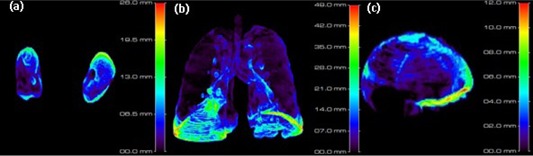
3D distance map for kidneys (a), lungs (b), and brain (c) for the worst case study.

As mentioned earlier, the Hausdorff distance is the maximum distance one would need to move the boundaries of the source region to completely cover the target region which is obtained by finding the maximum value of the distance map. Figure 5 shows the box and whisker plot of maximum and minimum distances for 28 patients. Detailed statistical parameters for Figs. 5 to 7 are summarized in Table 3.

For all measured organs, the Hausdorff distance is above 10 mm. In some cases computed Hausdorff distance for whole body exceeds 50 mm. This relatively high error originates from the interscan movements of patient arms. Due to the higher degree of freedom, arms are potential source of mismatch. In addition to arms, slight movements of the head bring about misregistration errors, as well. In comparison with arms, head misregistration happens seldom in patients. Therefore the computed Hausdorff distance shows smaller misregistration errors for the brain than for the whole body. Figure 6 illustrates the arms and head misregistration as the worst case.

**Table 3:** Statistical characteristics of results presented in Figs. 5 to 7 for grouped analysis of 28 clinical studies reporting volume and surface discrepancies of four anatomical regions. Δ is as defined in Table 2

		*Minimum*	*1st Quartile*	Median±Δ	*3rd Quartile*	*Maximum*
Whole body	Target Overlap	0.876	0.909	0.929±0.01	0.953	0.961
	Mean Overlap (DC)	0.831	0.851	0.884±0.02	0.913	0.935
	Union Overlap (JC)	0.908	0.919	0.939±0.01	0.954	0.966
	False Negative	0.013	0.035	0.049±0.01	0.054	0.082
	False Positive	0.039	0.047	0.071±0.01	0.091	0.124
	Volume Similarity Error	−0.040	−0.020	−0.012±0.01	−0.002	0.010
	Hausdorff Distance (mm)	56.7	75.8	106.8±14.5	123.0	153.8
	Distance Error (mm)	3.0	4.6	6.5±1.4	9.2	13.3
Brain	Target Overlap	0.607	0.821	0.906±0.05	0.975	0.996
	Mean Overlap (DC)	0.402	0.649	0.779±0.06	0.845	0.895
	Union Overlap (JC)	0.623	0.787	0.876±0.04	0.916	0.945
	False Negative	0.072	0.111	0.164±0.04	0.234	0.374
	False Positive	0.004	0.025	0.094±0.05	0.179	0.393
	Volume Similarity Error	−0.063	0.008	0.045±0.02	0.063	0.107
	Hausdorff distance (mm)	11.5	15.4	21.6±7.2	38.8	64.3
	Distance Error (mm)	1.4	2.6	3.7±1.3	6.7	8.3
Lungs	Target Overlap	0.613	0.800	0.867±0.04	0.926	0.969
	Mean Overlap (DC)	0.591	0.737	0.787±0.03	0.840	0.928
	Union Overlap (JC)	0.829	0.849	0.881±0.02	0.913	0.962
	False Negative	0.029	0.050	0.107±0.03	0.134	0.213
	False Positive	0.031	0.074	0.133±0.04	0.200	0.387
	Volume Similarity Error	−0.095	−0.054	−0.023±0.02	0.004	0.081
	Hausdorff Distance (mm)	20.9	26.0	36.2±4.3	39.8	53.6
	Distance Error (mm)	1.1	2.3	3.0±0.5	3.8	5.4
Kidneys	Target Overlap	0.431	0.574	0.715±0.07	0.798	0.916
	Mean Overlap (DC)	0.396	0.461	0.579±0.07	0.673	0.750
	Union Overlap (JC)	0.567	0.631	0.733±0.05	0.805	0.857
	False Negative	0.000	0.088	0.196±0.06	0.280	0.446
	False Positive	0.000	0.157	0.245±0.06	0.336	0.569
	Volume Similarity Error	−0.316	−0.108	−0.010±0.04	0.034	0.139
	Hausdorff Distance (mm)	0.0	14.7	17.3±3.5	26.0	32.9
	Distance Error (mm)	0.0	2.5	3.6±0.9	5.6	6.0

Apart from the local misregistration (in the arms), the distance error, which is the mean value of the distance map, indicates a fair agreement (under 10 mm) for the majority of patients (Fig. 5). Diaphragmatic function is the principal source of misregistration in the lungs and even kidneys. A close look at the 3D distance map of lungs reveals that the lungs’ base and the kidneys’ apex are the most susceptible to misregistration error (Fig. 7 (a)–(b).

## IV. DISCUSSION

In this work, we performed and evaluated a 3D whole‐body CT to MRI nonrigid image registration algorithm aiming at elaborating a framework for implementation and validation of MRI‐guided attenuation correction strategies on hybrid PET/MRI systems. The validation part relied on the use of retrospectively acquired clinical data and various metrics including qualitative visual assessment performed by an experienced physician and objective computer‐aided measurements. A limited number of studies focused on the use of 3D registration for MR‐guided attenuation correction.^41^, ^42^, ^43^ These contributions focused mostly on brain imaging applications where intramodality registration, even of a patient study to an atlas, is less challenging because of the rigidity of the head compared to whole‐body imaging. In addition, these contributions failed to describe in sufficient detail the registration procedures used to enable their reproduction and reuse and, more importantly, the registration accuracy was not assessed or quantified. One of these studies used an aligned MR/CT atlas to build a pseudo‐CT image using MR‐to‐MR registration (intramodality registration).^(41)^ More recently, the same group reported on the comparison of segmentation and atlas registration and pattern recognition‐based methods using whole‐body studies, and concluded that the latter provides better overall PET quantification accuracy than the MR image segmentation approach.^(11)^ The focus of this paper was on attenuation correction using the same registration approach. The visual assessment of registered images (Fig. 1) indicates excellent visual alignment of images after registration in most of the cases.

It is obvious that for the volume overlap metric (Fig. 3), the sensitivity of the measurement falls down as the volume increases. In other words, a tiny misalignment in small volumes will cause a greater effect on the global results. One can observe that we obtain higher correspondence for the whole body than kidneys. There is one case (patient 08) that falls under 0.3. This drastic falloff originates from the failure in detecting the kidneys owing to the lower image quality. As mentioned before, a DC (mean overlap) higher than 0.8 implies an excellent agreement of volumes. Thus, the majority of patients (above 75%) meet the criterion for whole body, brain, and lungs. On the other hand, for kidneys, the majority of patients satisfy the good agreement of volumes criteria (0.6≤DC<0.8). The smaller volume of the kidneys and the respiratory motion are the main causes of this reduction. Equation (5) explicitly demonstrates that the JC (union overlap) depends on the DC value. Therefore the discussion about DC backs up the JC, as well.

Comparing the false negative and false positive for the brain (Fig. 4) indicates a slightly higher false‐positive error. Owing to the fact that the anterior half of the brain is smaller than the posterior half, any anterior–posterior shift will bring about different false‐negative and false‐positive error. There is a fair agreement between the manual evaluation based on expert knowledge (Fig. 2) and objective evaluation based on computational figures of merit (Fig. 5) for lungs and kidneys.

The previously introduced quantities (Eq. (2–10)) are widely used in the evaluation of medical image registration and segmentation algorithms. For evaluation of image segmentation, manually segmented and automatically segmented objects are usually compared on the perfectly aligned images.^44^, ^45^, ^46^ Some studies reported Dice coefficients close to 0.7 as excellent results.^(46)^ Likewise, many papers used evaluation metrics to compare different types of registration algorithms.^25^, ^47^ In our work, we merely validated our intermodality deformable registration process to build a whole‐body CT‐MR atlas and define a framework for validation of MR‐guided attenuation correction in hybrid PET/MRI using CT‐based attenuation correction as reference.

We strived to cover the extensive range of quantities available in this study. Nevertheless, since the whole registration process is sensitive to geometry and differing from one object to the other, proposing a comprehensive approach for evaluation of deformable image registration is not an easy task, particularly for whole‐body imaging. Algorithms developed to address the registration problem for a specific body region (e.g., the lungs) and validated using special test cases for that region might not perform equally well in other regions of the body or in whole‐body studies. Therefore, we do not claim that our evaluation is complete and perfect, as it includes many limitations. The limited number of anatomic landmarks can be considered as the main limitation of our work. We aimed at selecting a largest number of data points per site for reliable evaluation. However, using a large number of data points per anatomical site is difficult to achieve when a human observer is involved. Another limitation is that our evaluation relied heavily on the segmentation of the whole body and three specific organs. The errors and uncertainties associated with segmentation may be considered as prevailing origin of the discrepancies. Segmentation errors also need to be estimated using a reproducibility experiment involving multiple observers over multiple time points, which was not possible in our case owing to the lack of qualified human observers willing to perform this tedious task. Nevertheless, it is commonplace to use human expert knowledge as ground truth for evaluation of image segmentation techniques. Indeed, most of the studies cited did not perform multiple observer studies owing to the above‐mentioned difficulties.

In addition to the above, when probing outliers from box and whisker plots, we encountered cases where the organ being considered is indistinguishable, which consequently causes registration and segmentation errors. The segmentation errors have several origins, including low image contrast in low‐resolution MR images used in this protocol, metal artifacts in images of some patients bearing metallic implants (patient #21), and respiratory motion artifacts.

There are limited data in the literature reporting on performance assessment of whole‐body multimodality image registration since most of the studies focused on specific regions of the body, mainly the brain where registration with high accuracy could be achieved. Our experience in cardiac perfusion PET/CT studies led to the conclusion that deformable registration outperforms both manual and rigid‐body registration.^(48)^ Klein et al.^(27)^ reported a mean distance between corresponding points of 1 mm for brain imaging using elastix. Our experience with elastix for deformable atlas registration in small animal PET/CT imaging revealed a mean registration mismatch distance of about 6 mm with good quantification accuracy in most of the regions, especially the brain, but not in the bladder.^(49)^ In this work, a median distance error of 3.7 mm was obtained for brain segmentation (Table 3), which is reasonable for a whole‐body registration. The median of our registration errors for anatomic landmarks varies between 7 mm and 14 mm; however, the misregistrations were local. A lower overall error can be expected in our case. It should be noted that because of the retrospective nature of the study, physical spacing along Z direction for the datasets used in this work is much larger than that of axial (instead of equal physical spacing), which might affect the registration accuracy. Uniform sampling in physical space is more effective than uniform sampling in voxel space (in case voxels are not isomeric). However, even in this case we did not consider all control points. Eight‐voxel spacing will result in over 30,000 control points, which will considerably slow down the whole process. Our registration procedure randomly selects 6,000 control points for each iteration.

Segmentation of all organs in both CT and MR images is tricky, and on that account we considered only three organs located in different regions of the body to obtain a global estimation of the performance of the image registration algorithm. Various patient motions, such as physiological respiratory motion and arms motion, are other sources of error that can compromise the registration process.

The registered image pairs constitute a useful 3D whole‐body MR‐CT atlas, suitable for the development and evaluation of novel MR‐guided attenuation correction procedures for hybrid PET/MRI systems. Our aim is to assess the potential of various knowledge base learning strategies to convert MR to pseudo‐CT images, enabling the generation of attenuation maps with continuous variation in attenuation correction factors through classification of bone and consideration of lung inhomogeneities. This work is under progress and will be reported in future publications.

## V. CONCLUSIONS

We have developed and evaluated an MR to CT deformable registration algorithm using 28 clinical whole‐body scans. The registration could be performed from CT to MR space either by applying inverse transform parameters or performing another registration. Our intention in this work was to assess the performance of the registration algorithm while keeping original CT with defined landmarks as reference. The registration of large volumes requires more efforts in terms of computing time and computational complexity. Although some of the considered evaluation metrics (DC and JC) were not independent, we deliberately included all potential metrics for evaluation. Our 3D registration process suffers from mismatch in the arms in some cases. However, the applied method is able to accurately register the arms and other parts of the body in most of the cases at the expense of computation time. Besides, for the targeted objective (evaluation and atlas generation for MR‐guided attenuation correction), the lack of correspondence at the level of the arms will not be a major obstacle.

## ACKNOWLEDGMENTS

This work was supported by the Swiss National Science Foundation under grants SNSF 31003A‐125246 and 33CM30‐124114, Geneva Cancer League, and Tehran University of Medical Sciences under grant No. 89‐02‐30‐10934.

## References

[1] Fritsch DS , Chaney EL , Boxwala A et al. Core‐based portal image registration for automatic radiotherapy treatment verification. Int J Radiat Oncol Biol Phys. 1995;33(5):1287–300. 7493854 10.1016/0360-3016(95)02092-6

[2] Kunzler T , Grezdo J , Bogner J , Birkfellner W , Georg D . Registration of DRRs and portal images for verification of stereotactic body radiotherapy: a feasibility study in lung cancer treatment. Phys Med Biol. 2007;52(8):2157–70. 17404461 10.1088/0031-9155/52/8/008

[3] Lewis PJ , Siegel A , Siegel AM et al. Does performing image registration and subtraction in ictal brain SPECT help localize neocortical seizures? J Nucl Med. 2000;41(10):1619–26. 11037989

[4] Mumcuoglu EU , Nar F , Yardimci Y et al. Simultaneous surface registration of ictal and interictal SPECT and magnetic resonance images for epilepsy studies. Nucl Med Commun. 2006;27(1):45–55. 16340723 10.1097/01.mnm.0000189775.75743.0b

[5] Wang Q , Nicholson PH , Timonen J et al. Monitoring bone growth using quantitative ultrasound in comparison with DXA and pQCT. J Clin Densitom. 2008;11(2):295–301. 18158265 10.1016/j.jocd.2007.10.003

[6] Kyriacou SK , Davatzikos C , Zinreich SJ , Bryan RN . Nonlinear elastic registration of brain images with tumor pathology using a biomechanical model. IEEE Trans Med Imaging. 1999;18(7):580–92. 10504092 10.1109/42.790458

[7] De Moor K , Nuyts J , Plessers L , Stroobants S , Maes F , Dupont P . Non‐rigid registration with position dependent rigidity for whole body PET follow‐up studies. Proc IEEE Nuclear Science Symposium and Medical Imaging Conference, 2006. Piscataway, NJ: IEEE; 2006 p. 3502–06.

[8] Christensen GE , Song JH , Lu W , El Naqa I , Low DA . Tracking lung tissue motion and expansion/compression with inverse consistent image registration and spirometry. Med Phys. 2007;34(6):2155–63. 17654918 10.1118/1.2731029

[9] Wu C , Murtha PE , Jaramaz B . Femur statistical atlas construction based on two‐level 3D non‐rigid registration. Comput Aided Surg. 2009;14(4–6):83–99. 20121588 10.3109/10929080903246543

[10] Hardisty M , Gordon L , Agarwal P , Skrinskas T , Whyne C . Quantitative characterization of metastatic disease in the spine. Part I. Semiautomated segmentation using atlas‐based deformable registration and the level set method. Med Phys. 2007;34(8):3127–34. 17879773 10.1118/1.2746498

[11] Hofmann M , Bezrukov I , Mantlik F et al. MRI‐based attenuation correction for whole‐body PET/MRI: quantitative evaluation of segmentation‐ and atlas‐based methods. J Nucl Med. 2011;52(9):1392–99. 21828115 10.2967/jnumed.110.078949

[12] Manenti G , Ciccio C , Squillaci E et al. Role of combined DWIBS/3D‐CE‐T1w whole‐body MRI in tumor staging: comparison with PET‐CT. Eur J Radiol. 2012;81(8):1917–25. 21908120 10.1016/j.ejrad.2011.08.005

[13] Drzezga A , Souvatzoglou M , Eiber M et al. First clinical experience of integrated whole‐body PET/MR. Comparison to PET/CT in patients with oncological diagnoses. J Nucl Med. 2012;53(6):845–55. 22534830 10.2967/jnumed.111.098608

[14] Ng SH , Chan SC , Yen TC et al. PET/CT and 3‐T whole‐body MRI in the detection of malignancy in treated oropharyngeal and hypopharyngeal carcinoma. Eur J Nucl Med Mol Imaging. 2011;38(6):996–1008. 21327634 10.1007/s00259-011-1740-1

[15] Zaidi H , Montandon ML , Alavi A . The clinical role of fusion imaging using PET, CT, and MR imaging. Magn Reson Imaging Clin N Am. 2010;18(1):133–49. 19962098 10.1016/j.mric.2009.09.010

[16] Heiss WD . The potential of PET/MR for brain imaging. Eur J Nucl Med Mol Imaging. 2009;36(Suppl 1):S105–S112. 19104801 10.1007/s00259-008-0962-3

[17] Antoch G and Bockisch A . Combined PET/MRI: a new dimension in whole‐body oncology imaging? Eur J Nucl Med Mol Imaging. 2009;36(Suppl 1):S113–S120. 19104802 10.1007/s00259-008-0951-6

[18] Zaidi H and Del Guerra A . An outlook on future designs of hybrid PET/MRI systems. Med Phys. 2011;38(10):5667–89. 21992383 10.1118/1.3633909

[19] Maintz JB and Viergever MA . A survey of medical image registration. Med Image Anal. 1998;2(1):1–36. 10638851 10.1016/s1361-8415(01)80026-8

[20] Vandemeulebroucke J , Rit S , Kybic J , Clarysse P , Sarrut D . Spatiotemporal motion estimation for respiratory‐correlated imaging of the lungs. Med Phys. 2011;38(1):166–78. 21361185 10.1118/1.3523619

[21] Murphy K , van Ginneken B , Reinhardt JM et al. Evaluation of registration methods on thoracic CT: the EMPIRE10 challenge. IEEE Trans Med Imaging. 2011;30(11):1901–20. 21632295 10.1109/TMI.2011.2158349

[22] Zhong H , Kim J , Chetty IJ . Analysis of deformable image registration accuracy using computational modeling. Med Phys. 2010;37(3):970–79. 20384233 10.1118/1.3302141 PMC3188658

[23] Bender ET and Tomé WA . The utilization of consistency metrics for error analysis in deformable image registration. Phys Med Biol. 2009;54(18):5561–77. 19717890 10.1088/0031-9155/54/18/014 PMC2798737

[24] Wang H , Dong L , O'Daniel J et al. Validation of an accelerated ‘demons’ algorithm for deformable image registration in radiation therapy. Phys Med Biol. 2005;50(12):2887–905. 15930609 10.1088/0031-9155/50/12/011

[25] Klein A , Andersson J , Ardekani BA et al. Evaluation of 14 nonlinear deformation algorithms applied to human brain MRI registration. Neuroimage. 2009;46(3):786–802. 19195496 10.1016/j.neuroimage.2008.12.037 PMC2747506

[26] Rueckert D , Sonoda LI , Hayes C , Hill DLG , Leach MO , Hawkes DJ . Nonrigid registration using free‐form deformations: application to breast MR images. IEEE Trans Med Imaging. 1999;18(8):712–21. 10534053 10.1109/42.796284

[27] Klein S , Staring M , Murphy K , Viergever MA , Pluim JPW . Elastix: a toolbox for intensity‐based medical image registration. IEEE Trans Med Imaging. 2010;29(1):196–205. 19923044 10.1109/TMI.2009.2035616

[28] Yoo TS , Ackerman MJ , Lorensen WE et al. Engineering and algorithm design for an image processing Api: a technical report on ITK‐the Insight Toolkit. Stud Health Technol Inform. 2002;85:586–92. 15458157

[29] Murphy K , van Ginneken B , Pluim JP , Klein S , Staring M . Semi‐automatic reference standard construction for quantitative evaluation of lung CT registration. Med Image Comput Assist Interv. 2008;11(Pt 2):1006–13. 10.1007/978-3-540-85990-1_121 18982703

[30] Klein S , Staring M , Pluim JPW . Evaluation of optimization methods for nonrigid medical image registration using mutual information and B‐splines. IEEE Trans Image Process 2007;16(12):2879–90. 18092588 10.1109/tip.2007.909412

[31] Zaidi H , Ojha N , Morich M et al. Design and performance evaluation of a whole‐body Ingenuity TF PET‐MRI system. Phys Med Biol. 2011;56(10):3091–106. 21508443 10.1088/0031-9155/56/10/013 PMC4059360

[32] Donati OF , Reiner CS , Hany TF et al. 18F‐FDG‐PET and MRI in patients with malignancies of the liver and pancreas. Accuracy of retrospective multimodality image registration by using the CT‐component of PET/CT. Nuklearmedizin. 2010;49(3):106–14. 20407733 10.3413/nukmed-0263

[33] Grgic A , Nestle U , Schaefer‐Schuler A et al. Nonrigid versus rigid registration of thoracic 18F‐FDG PET and CT in patients with lung cancer: an intraindividual comparison of different breathing maneuvers. J Nucl Med. 2009;50(12):1921–26. 19910420 10.2967/jnumed.109.065649

[34] Yushkevich PA , Piven J , Hazlett HC et al. User‐guided 3D active contour segmentation of anatomical structures: significantly improved efficiency and reliability. Neuroimage. 2006;31(3):1116–28. 16545965 10.1016/j.neuroimage.2006.01.015

[35] Crum WR , Camara O , Hill DL . Generalized overlap measures for evaluation and validation in medical image analysis. IEEE Trans Med Imaging. 2006;25(11):1451–61. 17117774 10.1109/TMI.2006.880587

[36] Dice LR . Measures of the amount of ecologic association between species. Ecology. 1945;26(3):297–302.

[37] Zijdenbos AP , Dawant BM , Margolin RA , Palmer AC . Morphometric analysis of white matter lesions in MR images: method and validation. IEEE Trans Med Imaging. 1994;13(4):716–24. 18218550 10.1109/42.363096

[38] Fleiss JL , Levin B , Paik MC . Statistical methods for rates and proportions, 3^rd^ edition. Hoboken, NJ: Wiley; 2003.

[39] Jaccard P . The distribution of the flora in the Alpine zone. New Phytologist. 1912;11(2):37–50.

[40] Duda R and Hart P . Pattern classification and scene analysis. Hoboken, NJ: Wiley‐Interscience Publication; 1973.

[41] Hofmann M , Steinke F , Scheel V et al. MRI‐based attenuation correction for PET/MRI: a novel approach combining pattern recognition and atlas registration. J Nucl Med. 2008;49(11):1875–83. 18927326 10.2967/jnumed.107.049353

[42] Fei B , Yang X , Nye JA et al. MR/PET quantification tools: registration, segmentation, classification, and MR‐based attenuation correction. Med Phys. 2012;39(10):6443–54. 23039679 10.1118/1.4754796 PMC3477199

[43] Schreibmann E , Nye JA , Schuster DM , Martin DR , Votaw J , Fox T . MR‐based attenuation correction for hybrid PET‐MR brain imaging systems using deformable image registration. Med Phys. 2010;37(5):2101–09. 20527543 10.1118/1.3377774

[44] Kirisli HA , Schaap M , Klein S et al. Evaluation of a multi‐atlas based method for segmentation of cardiac CTA data: a large‐scale, multicenter, and multivendor study. Med Phys. 2010;37(12):6279–91. 21302784 10.1118/1.3512795

[45] Stapleford LJ , Lawson JD , Perkins C et al. Evaluation of automatic atlas‐based lymph node segmentation for head‐and‐neck cancer. Int J Radiat Oncol Biol Phys. 2010;77(3):959–66. 20231069 10.1016/j.ijrobp.2009.09.023

[46] Pasquier D , Lacornerie T , Vermandel M , Rousseau J , Lartigau E , Betrouni N . Automatic segmentation of pelvic structures from magnetic resonance images for prostate cancer radiotherapy. Int J Radiat Oncol Biol Phys. 2007;68(2):592–600. 17498571 10.1016/j.ijrobp.2007.02.005

[47] Castadot P , Lee JA , Parraga A , Geets X , Macq B , Grégoire V . Comparison of 12 deformable registration strategies in adaptive radiation therapy for the treatment of head and neck tumors. Radiother Oncol. 2008;89(1):1–12. 18501456 10.1016/j.radonc.2008.04.010

[48] Zaidi H , Nkoulou R , Baskin A et al. Computed tomography calcium score scan for attenuation correction of N‐13 ammonia cardiac positron emission tomography: effect of respiratory phase and registration method. Int J Cardiovasc Imaging. [In press] 2013. Mar 17. [Epub ahead of print] 10.1007/s10554-013-0207-9 23504215

[49] Gutierrez D and Zaidi H . Automated analysis of small animal PET studies through deformable registration to an atlas. Eur J Nucl Med Mol Imaging. 2012;39(11):1807–20. 22820650 10.1007/s00259-012-2188-7 PMC3464388

